# Understanding Medical Education: Evidence, Theory and Practice (2nd edn)

**DOI:** 10.1192/pb.bp.114.047795

**Published:** 2015-04

**Authors:** Brian Lunn

**Figure:**
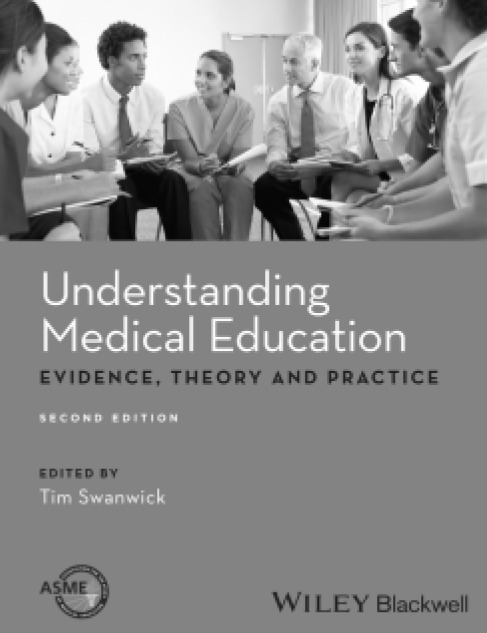


Why should a jobbing psychiatrist be interested in a medical education textbook? Simply put, it is that we have shifted from an era of ‘See one, do one, teach one’ to one where an evidence base is available for education, not just for treatments. This is particularly the case for medical education, where the need to evidence pedagogic practice for regulators has been a priority for many years and has driven research to support and drive practice. This is an increasingly important issue. The General Medical Council, in *Recognition and Approval of Trainers*, has set out the standards it requires for clinicians to be trainers, with an implementation date of 31 July 2016, and a similar standardised approval process is well underway for undergraduate teaching.

*Understanding Medical Education* is a good place to start for those wishing to build their knowledge of the evidence base to inform their teaching. With a strong cast of the ‘usual suspects’ in the field, it delivers a broad range of chapters covering the breadth of educational topics. The book is set out in themed sections allowing selection of topics of interest. For the determined reader, read sequentially they build from basic foundations through strategy and assessment to research and finally, a ‘Staff and Students’ section that covers issues related to learners and teachers. This all finishes with an excellent chapter on educational leadership.

This should not be seen as a book targeted at the academic community. Even though one or two chapters may not affect most doctors’ teaching practice (e.g. the chapter on curriculum design), they will nonetheless enhance understanding of the choices that went into learning and teaching strategies.

So at the end of this, a reasonable question might be, ‘Is it worth my time buying/borrowing this book?’ If you already have a strong background in pedagogic theory, then it gives up-to-date monographs collected together in one place but perhaps nothing new, so it may be one you borrow rather than buy (and this is not a criticism). If you are developing your knowledge or wish a reference to support your teaching, whether undergraduate or postgraduate, then the answer is indubitably ‘Yes.’

